# Smart probes for optical imaging of T cells and screening of anti-cancer immunotherapies

**DOI:** 10.1039/d2cs00928e

**Published:** 2023-06-28

**Authors:** Marco Bertolini, Man Sing Wong, Lorena Mendive-Tapia, Marc Vendrell

**Affiliations:** a Centre for Inflammation Research, The University of Edinburgh EH16 4UU Edinburgh UK marc.vendrell@ed.ac.uk; b IRR Chemistry Hub, Institute for Regeneration and Repair, The University of Edinburgh EH16 4UU Edinburgh UK

## Abstract

T cells are an essential part of the immune system with crucial roles in adaptive response and the maintenance of tissue homeostasis. Depending on their microenvironment, T cells can be differentiated into multiple states with distinct functions. This myriad of cellular activities have prompted the development of numerous smart probes, ranging from small molecule fluorophores to nanoconstructs with variable molecular architectures and fluorescence emission mechanisms. In this Tutorial Review, we summarize recent efforts in the design, synthesis and application of smart probes for imaging T cells in tumors and inflammation sites by targeting metabolic and enzymatic biomarkers as well as specific surface receptors. Finally, we briefly review current strategies for how smart probes are employed to monitor the response of T cells to anti-cancer immunotherapies. We hope that this Review may help chemists, biologists and immunologists to design the next generation of molecular imaging probes for T cells and anti-cancer immunotherapies.

## Abstract 2

T cells are an essential component of the immune system and display multiple biological functions. Smart probes range from small fluorophores to nanoconstructs, and can target metabolic and enzymatic biomarkers as well as cell-surface receptors.

## Graphical abstract

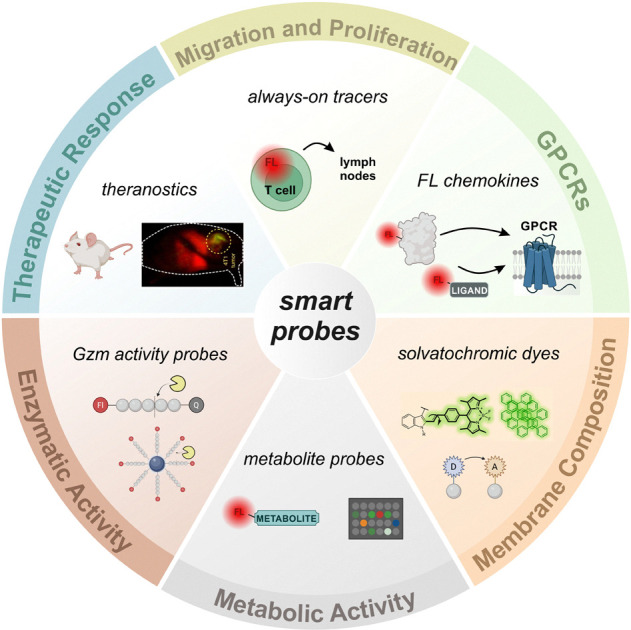

**Key learning points**

(1) Chemical strategies to build activatable probes for imaging T cell activity.

(2) Advantages and limitations of smart chemical constructs using different activation mechanisms (*e.g.*, receptor-bound, metabolically-activated, enzyme-triggered).

(3) Druggable biological targets associated with T cell activation and exhaustion.

(4) Molecular imaging approaches for fluorescence-based monitoring of response to anti-cancer immunotherapies.

(5) Perspectives and future challenges for translational imaging in clinical studies.

## 1. Introduction

**Fig. 1:**
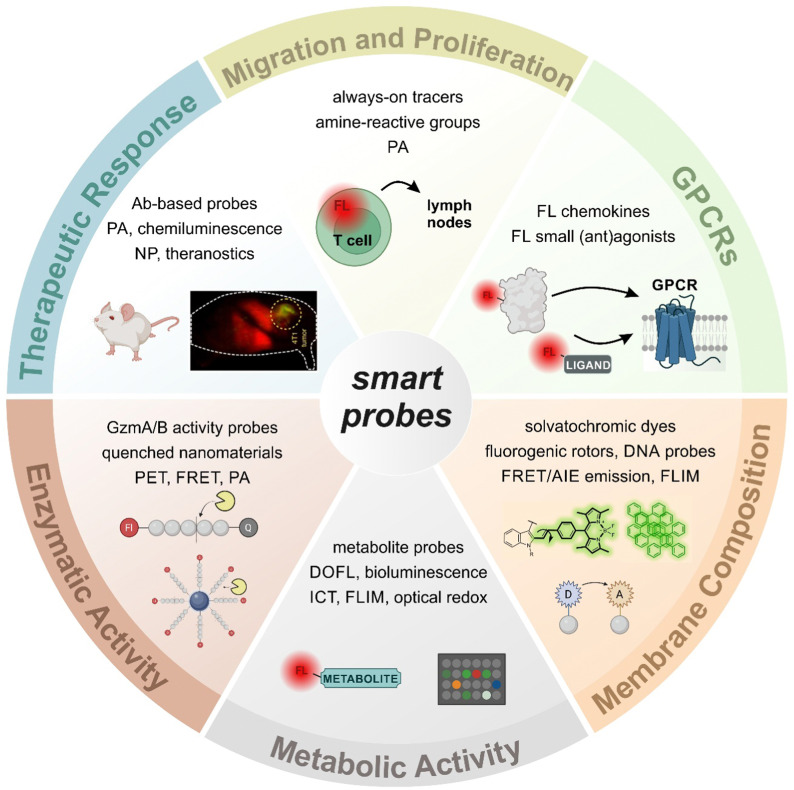
A plethora of smart architectures to image T cell activity. The involvement of T lymphocytes in both physiological and pathological conditions has led to the development of a broad variety of smart probes for *in vitro* and *in vivo* imaging. Smart probes can be activated by a wide range of stimuli (*e.g.*, enzymatic activity, membrane fluidity, metabolite production or receptor expression) that are associated with T cell function, such as proliferation, migration, and cytotoxic activity. Abbreviations: Ab: antibody, AIE: aggregation-induced emission, DOFL: diversity-oriented fluorescence library, FL: fluorescent, FLIM: fluorescence lifetime imaging microscopy, FRET: Förster resonance energy transfer, GPCR: G protein-coupled receptor, Gzm: granzyme, ICT: intramolecular charge transfer, NP: nanoparticle, PA: photoacoustic, PET: positron emission tomography.

Fluorescence imaging has revolutionized the analysis of biological systems by allowing the visualization of molecular events with unprecedented sensitivity and resolution. Although the development of photostable and bright fluorescent probes has opened the possibility of imaging cells in real time, the information that can be obtained from “always-on” fluorophores can be limited. Recent advancements in fluorophore chemistry have rendered activatable fluorophores -also known as smart probes-, whose emission is triggered by specific stimuli, such as metabolites, pH or enzymatic activity.^1–6^ Other smart chemical designs rely on targeting moieties that deliver fluorescent payloads in close proximity to proteins and receptors exposed on the cellular membrane.^7,8^ Activatable fluorophores often exhibit higher signal-to-noise ratios than “always-on” fluorophores, making them ideal for monitoring cellular function over time.^9–11^ In addition to these favorable properties, smart probes can be also fine-tuned to display excitation and emission wavelengths in the near-infrared (NIR) region, where absorption and autofluorescence of endogenous biomolecules are minimal.^12–14^ Notably, new technologies such as photoacoustic (PA) and fluorescence lifetime imaging (FLIM) are emerging as promising techniques due to their intrinsic high contrast and multiplexing capabilities.^15–17^ The features of smart fluorescent architectures provide a wide range of possibilities in biomedical research, from the interrogation of functional states *in vitro* to the *in vivo* evaluation of anti-cancer immunotherapies in preclinical models (Fig. 1).^18,19^

**Fig. 2:**
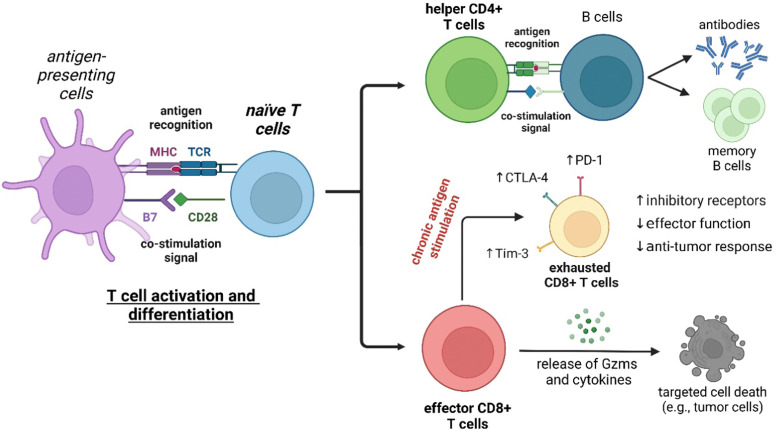
T cell development and regulation of immune function. Naïve CD4+ or CD8+ T cells are activated and differentiated upon interaction of the membrane T cell receptors (TCRs) with major histocompatibility complex (MHC) molecules and co-stimulatory signals from antigen-presenting cells. Helper CD4+ T cells play crucial roles in activating B cells to produce antibodies for specific pathogens. Additionally, the activation of helper CD4+ T cells also leads to the generation of memory B cells that produce a rapid secondary immune response after exposure to the same antigen. On the other hand, effector CD8+ T cells display cytotoxic activity on viral-infected or cancer cells through the secretion of granzymes and cytokines that cause cell death. However, chronic antigen stimulation can result in CD8+ T cell exhaustion, which is characterized by the overexpression of inhibitory receptors such as PD-1, CTLA-4, and Tim-3 and the consequent loss of anti-tumour response and effector functions.

T lymphocytes are key players of the adaptive immune system and essential for the homeostasis of both cellular and humoral immunity (Fig. 2). Cytotoxic CD8+ T cells are a crucial component of the anti-tumoral response by releasing proteins (*e.g.*, perforin, granzymes) that can lead to cancer cell death, while helper CD4+ T cells stimulate the production of antigen-specific antibodies.^20^ However, the activation of T cells is not always beneficial as they can also contribute to the onset of autoimmune diseases, such as rheumatoid arthritis and psoriasis, where the immune system is abnormally activated and causes extensive damage to healthy tissues.^21^ The broad repertoire of roles that T cells play in health and disease make them an appealing target for the smart probe development in order to generate molecules that can selectively track their mobilization and phenotype. Furthermore, T cell-mediated responses play a critical role in anti-cancer immunotherapies, with immune checkpoint inhibitors and chimeric antigen receptor T cells (CAR T cells) being promising therapeutic strategies to stimulate or reprogram their ability to kill cancer cells.^22,23^ In the recent years, smart probes have been harnessed to directly evaluate the efficacy of such treatments without the need for invasive techniques (*e.g.*, tumor biopsies) and also been adapted to high-throughput screening platforms to optimize combination therapies.

In this review, we will present the latest advances in the chemical design and applications of fluorescent smart probes for imaging the activity of T cells. With a few exceptions, our article does not primarily cover the design and application of genetically encoded reporters, because these have been reviewed elsewhere;^24^ instead, we will focus on the molecular design of fluorescent conjugates combining optical reporters and biomolecular units (*e.g.*, small molecules, peptides, proteins, nanomaterials) that target multiple aspects of T cell biology (*e.g.*, metabolism, expression of T cell receptors, enzymatic activity). Furthermore, the review will also cover examples of how T cell-targeted smart probes have been applied to monitor the efficacy of anti-cancer immunotherapies, including a variety of optical imaging modalities and preclinical models.

## 2. T cell migration tracers and probes targeting T cell metabolism

The migration of T cells to tumor sites is crucial to achieve effective immune responses against cancer cells. In the complex tumor microenvironment (TME), specific cell–cell interactions and environmental factors define the proliferation, metabolism and function of T cells.^25–27^ Therefore, the design of imaging agents that can monitor the migration, biodistribution and activity of T cells is needed to optimize the efficacy of cell transfer therapies. Current methods to characterize T cell subtypes at inflammation sites typically rely on antibody-based methods to trace the expression of cell surface proteins and/or cytokine production;^28^ however, antibodies have limited permeability and tissue accessibility as well as slow kinetics, which hamper real-time imaging of intact live cells and tissues. Therefore, there is a demand for alternative strategies using smaller fluorescent probes that can be used to phenotype the functional state of T cells in clinical samples in real time. Smart fluorescent probes can also be chemically fine-tuned to selectively image subsets of T cells with enhanced sensitivity. In this section, we will discuss the chemical design of fluorescent probes to image the migration, proliferation and metabolic reprogramming of T cells.

### 2.1. Fluorescence tracking of T lymphocyte migration and proliferation

Some of the first chemical designs for imaging T cell in inflamed tissues were based on non-specific, cell-permeable fluorophores. For example, the group of Weissleder prepared a far-red fluorescent probe (*i.e.*, VT680) bearing an amine reactive *N*-hydroxysuccinimide (NHS) group that could enter T cells and covalently bind to cellular components without leaking into adjacent cells.^29^ In experiments with HA-bearing tumor mouse models of adoptive transfer immunotherapy, VT680-labeled HA-specific cytotoxic T lymphocytes (CTLs) were injected into mice to monitor their biodistribution and accumulation in tumors and lymph nodes. The fluorescence signals of VT680-labeled T cells were quantified at single-cell level and for several days post injection. Similarly, Foster *et al.* reported the NIR IRDye800CW as another NHS-containing reactive fluorophore for monitoring the migration of CD3-activated human T cells in mice bearing subcutaneous tumors.^30^ In these experiments, tumors were modified to express the chemokine C–C Chemokine Ligand 5 (CCL5), which is a potent chemoattractant for immune cells. Increased fluorescence signals from IRDye800CW confirmed stronger infiltration of T cells at the tumor site when CCL5 was present in the TME when compared to control tumors. On the other hand, the authors also observed a rapid decrease in the signal from dividing cells, which limited its application for long-term imaging to assess proliferation or division indexes by fluorescence. This could be due to the negatively charged structure of IRDye800CW NHS, which favored the reaction with extracellular amino groups found on surface proteins, as shown by the fluorescence emission found around the cell membranes. In contrast, the cell permeable VT680 probe accumulated not only in the cell membrane but also in the cytoplasm and nucleus of T cells.

**Fig. 3:**
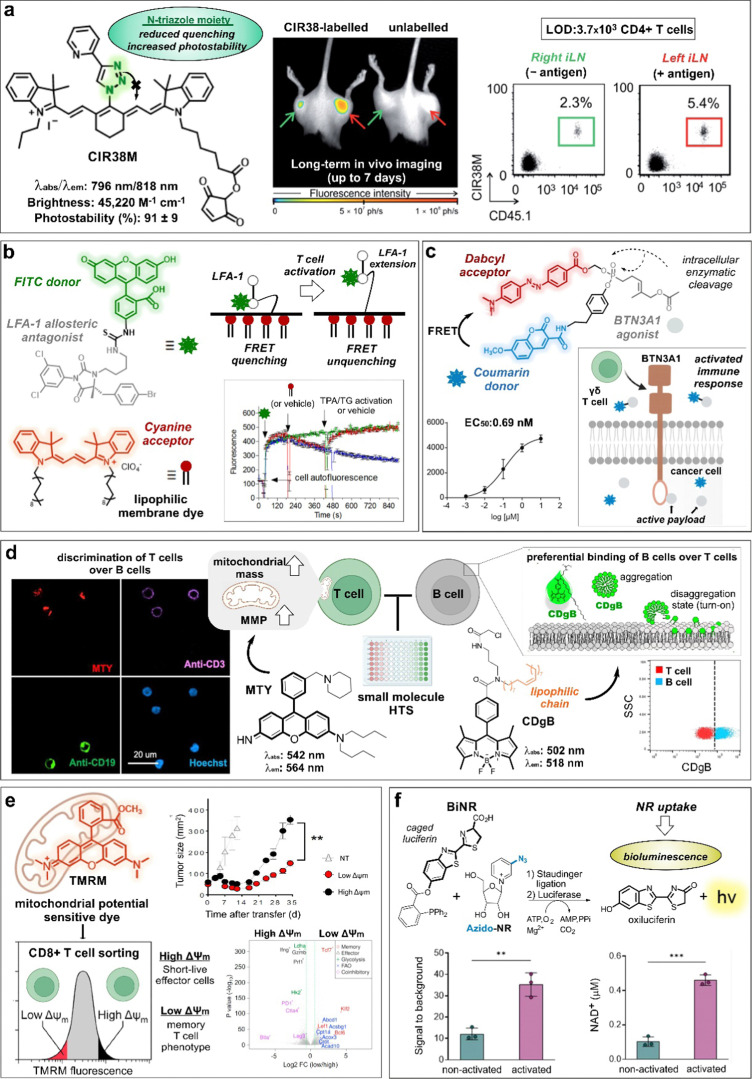
Smart probes targeting T cell metabolism and migration tracers. (a) Chemical structure and photophysical properties of the CIR38M tracer. In whole-body fluorescence imaging of mice injected with CIR38M-labeled (left) or unlabeled T cells (right), labeled cells selectively accumulate in the left hind legs (red arrow) where the antigen was administered. *Ex vivo* analysis of inguinal lymph nodes (ILNs) confirmed the migration of labeled cells to the left limb. (b) Chemical structures and activation mechanism of the LFA-1 specific FRET donor (BIRT-FITC) and its cyanine lipophilic acceptor. Activation of cells with phorbol ester (TPA) and thapsigargin (TG) resulted in LFA-1 extension, leading to FRET unquenching and increased FITC signal. (c) Structure of FRET probe, incorporating coumarin as the donor and Dabcyl as the acceptor quencher. Enzymatic cleavage of the phosphonate bond triggers the release of the quencher and the BTN3A-1 agonist, resulting in a dose dependent increase in fluorescence intensity and cell activation. Reproduced from ref. 34 with permission from Elsevier, copyright 2021. (d) Chemical structures of T and B cell specific probes derived from diversity-oriented libraries. MTY preferentially stained T cells (CD3 +) over B cells (CD19 +) in an *in vitro* assay, due to their high mitochondrial membrane potential (MMP). Probe CDgB selectively labeled the membrane of B cells over T cells in a flow cytometry experiment. Reproduced with permission from the American Chemical Society.^44^ (e) TMRM, a mitochondrial potential sensor, was used to sort high and low MMP T cells. These populations were transferred into tumor-bearing mice, and their tumor size was measured over time. The expression profiles of high and low MMP populations were analyzed and represented in a volcano plot. Reproduced with permission from Cell Press.^45^ (f) Chemical reaction between bioluminescent probe BiNR and azido-NR. BiNR activation was measured in stimulated and non-stimulated human T cells. This finding well correlated with the level of NAD+ present in activated vs non-activated CD3+ T cells. Reproduced from ref. 47 with permission from Elsevier, copyright 2023.

Considering the unmet need for chemical tools to monitor the outcome of immunotherapies, more sophisticated and versatile constructs have been designed in the last few years. The group of Fu and Nie reported the agent NIR-797 isothiocyanate for both fluorescent and photoacoustic monitoring of T cells, allowing much deeper penetration depth (up to 9.5 mm) for long-term imaging.^31^ In this case, the authors imaged the migration and proliferation of post-transferred T cells to lymph nodes, bacterial infection sites and tumors, observing variable levels of T cells across the different sites at different time points. Our group also reported the rational design of CIR38M as a NIR tricarbocyanine *N*-triazole probe for extended long-term *in vivo* imaging with enhanced brightness and photostability properties over previously reported tricarbocyanine dyes (Fig. 3a).^32^ In a mouse model of T cell activation by antigen-driven accumulation, the thiol-reactive CIR38M formed covalent bonds with intracellular proteins to allow monitoring of the fate of therapeutic CD4+ T cells in lymph nodes *in vivo*. Thanks to its neutral uncharged character, CIR38M displayed good solubility, cell permeability and marginal leakage to neighbouring cells, with only a small decline of fluorescence emission after several cell divisions. CIR38M was able to track small number of T cells (*e.g.*, in the range of 4000 cells) for up to 7 days, outperforming previous NIR dyes (*e.g.*, IRDye800CW NHS).

### 2.2. Fluorescent reporters of T lymphocyte activation and metabolic reprogramming

Recent efforts on T cell imaging have been aimed at not only detecting the presence of T lymphocytes but also their functional state. Chigaev *et al.* reported Forster resonance energy transfer (FRET) probes targeting conformational changes of lymphocyte function–associated antigen 1 (LFA-1), an important integrin for the communication between immune cells and other antigen-presenting cells (Fig. 3b).^33^ These probes were based on LFA-1 allosteric antagonists bearing a fluorescein isothiocyanate (FITC) unit as a donor and a lipid acceptor probe. With this design, T cell activation led to the extension of LFA-1 conformation and unquenching of the FRET signal. Alternatively, FRET probes have been also exploited to monitor payload release after cellular uptake for the modulation of T cell activity. Wiemer *et al.* combined a coumarin-Dabcyl FRET pair with a phosphonate-cleavable moiety to release an agonist ligand of the transmembrane protein BTN3A1 upon metabolic activation of γδ T cells (Fig. 3c).^34^ Notably, when the probe was added to K562 leukaemia cells and subsequently cultured with γδ T cells, the cleavage of the ligand cargo promoted cytokine production by γδ T cells (EC_50_: 0.69 nM). In another example of phosphonate-mediated cleavage, Wiemer *et al.* reported a similar coumarin diphosphate analogue to release a prodrug upon internalization to stimulate the immune response of human Vg9Vd2 T cells.^34^ Again, K562 cells pre-loaded with the probe were able to trigger Vg9Vd2 T cell-mediated killing (EC_50_: 180 nM).

After activation, T cells experience multiple metabolic changes to allow survival, proliferation, and effector function. Some of these changes involve the modulation of signaling pathways and metabolite uptake. For instance, cysteine incorporation to regulate glutathione levels is known to be increased in activated T cells to remove the excess of reactive oxygen species (ROS).^35,36^ In this context, Siska *et al.* reported a cystine-FITC probe to study the upregulation of the cystine transporter xCT (SLC7a11) in activated CD4+ and CD8+ human T cells.^37^ Although not very significant, higher cystine-FITC intracellular signals were detected in stimulated CD8+ T cells relative to CD4+ T cells, enabling to differentiate cystine uptake across different lymphocyte subpopulations. Fatty acids can also regulate the functional state of T cells in the TME. Whereas energy production on activated T cells is predominantly based on aerobic glycolysis, regulatory T cells (Tregs) rely on fatty acid oxidation pathways. Lesniak and co-workers reported quantum dot conjugates with BODIPY fluorophores and fatty acids to monitor the uptake of lipids in T cells *in vivo*.^38^ The authors found that fatty acids were predominantly taken up by CD4+ Foxp3+ Tregs when compared to effector T cell subsets in tumors, thus validating the role of fatty acid metabolism in Tregs. Nitric oxide (NO) is an important signaling intermediate in T cell activation known to regulate mitochondrial and cytoplasmic Ca^2+^ signals.^39^ In another example of cellular uptake, Vorob’ev *et al.* described the preparation of an acridone-based probe for sensing NO in human Jurkat T cells.^40^ In this case, the reaction between the smart probe and NO yielded a triazole derivative with increased fluorescence -due to blockage of photoinduced electron transfer (PeT)- and an hypsochromic shift in the emission wavelength (*e.g.*, from 564 nm to 495 nm). Metabolism-associated enzymes are also attractive targets for imaging T cells. Wang and colleagues reported the red-emissive cell-permeable DDPB probe with turn-on fluorescence response upon reaction with carboxylesterase-2 (CES2), which is associated with higher CD8+ T cell infiltration in the TME.^41,42^ The fluorescence activation mechanism relied on the enzymatic hydrolysis by CES2 to release a benzoyl group that suppressed the intramolecular charge transfer (ICT) quenching. The authors evaluated the smart probe in inactive and active T cells and tumor cells and showed that the fluorescence signals were higher in active T cells and correlated with the distribution of the enzyme.

Given that mitochondria are central organelles in cell metabolism, several groups have generated smart probes targeting mitochondrial features to identify subpopulations of T cells. Chang and co-workers reported the rosamine probe MTY, which preferentially stained the mitochondria of T cells over B cells due to their higher mitochondrial mass and membrane potential (MMP, Fig. 3d).^43^ MTY was identified from a high-throughput screening approach, where immune cells from peripheral blood were incubated with a diversity-oriented fluorescence library. Interestingly, the same authors had previously reported a BODIPY probe bearing a long fatty acid chain to preferentially accommodate in the membrane of B cells over T cells due to higher membrane flexibility (*i.e.*, shorter PCs, and low cholesterol content) (Fig. 3d).^44^ In the same line, the group of Sukumar and Restifo described a lipophilic cationic dye tetramethylrhodamine methyl ester (TMRM) for the isolation of CD8+ T cells with fatty acid oxidation-based metabolism by means of their distinct MMP (Fig. 3e).^45^ The authors found that CD8+ T cells with low levels of MMP were associated with long-term surveillance, expansion and enhanced anti-cancer activity when compared to T cells with higher levels of MMP. Interestingly, parallel genomic analyses have shown that low MMP levels correlate to a memory CD8+ T cell signature whereas higher MMP levels are linked to an effector T cell phenotype.

Nicotinamide adenine dinucleotide (NAD) is an important metabolic co-enzyme, with low levels of NAD being linked to different pathologies.^46^ Recently, Goun *et al.* reported a bioluminescent probe (BiNR) for *in vivo* monitoring of nicotinamide riboside uptake (Fig. 3f).^47^ BiNR employed a two-step process based on (1) the addition of a caged luciferin and (2) a subsequent Staudinger ligation with an azido-labeled metabolite to uncage the luciferin moiety. Using this approach, the bioluminescence emission after reaction with luciferase was directly proportional to the uptake of azido-modified metabolites. Notably, the authors observed that nicotinamide riboside uptake was significantly increased upon T cell activation, with 3-fold higher BiNR signals in activated human CD3+ T cells compared to metabolically less active naïve CD3+ T cells. Moreover, both helper CD4+ and cytotoxic CD8+ T cells exhibited 6-fold increase of nicotinamide riboside uptake on day 2-post activation relative to day 11, in line with a decreased stimulation effect. In another example, the group of Walsh and Skala^48^ used the fluorescence lifetime signals of the metabolic co-enzymes NAD(P)H and flavin adenine dinucleotide (FAD) as direct indicators of the redox and activation states of T cells. The authors used machine learning models to classify T cells according to their optical redox ratios (*i.e.*, NAD(P)H over NAD(P)H plus FAD) and found that active T cells displayed higher ratios than quiescent T cells.

## 3. Probes targeting T cell receptors

The interactions between T cells and other cells and signaling molecules that regulate T cell function is orchestrated by a plethora of cell-surface receptors (Fig. 2). Thus, T cell activation and differentiation is driven by the interaction of the membrane T cell receptor (TCR) cluster with major histocompatibility complex (MHC) molecules presented on the surface of antigen-presenting cells (APC). Moreover, effective TCR signaling also involves the engagement of additional co-stimulatory signals (*e.g.*, CD28) and the mutually exclusive T cell surface receptors CD8 and CD4. T cell inhibitory type receptors targeted by immune checkpoint inhibitors are other key contributors in regulating the immune response. Importantly, the upregulation of such receptors during chronic antigen stimulation is a known factor associated with T cell exhaustion. This section reviews fluorescent smart probes targeting a variety of T cell receptors, including G protein-coupled receptors (GPCRs) and immune checkpoint proteins as well as the composition and dynamics of the cell membrane to monitor and modulate antitumor immunity.

### 3.1. Fluorescent probes for live-cell imaging of GPCRs

GPCRs are the largest family of cell surface proteins, and transduce signaling pathways related to cell homeostasis and function.^49–52^ In particular, a variety of GPCRs expressed in T cells have been found to play key roles in regulating migration, activation and differentiation.^53,54^ Thus, dynamic changes in the GPCR expression levels on T cells are tightly linked to their biological roles, exhibiting different expression patterns depending on the activation state.^55^ Among these, the chemokine receptor CXCR4 is expressed in most leukocytes (*e.g.*, T lymphocytes) and its presence with its cognate ligand CXCL12 has also been found in metastatic tumors, regulating the infiltration of T cells. In early studies from Hatse *et al.*, the authors used an AlexaFluor647 (AF647)-CXCL12 conjugate to study CXCR4 expression in different subpopulations of lymphocytes, showing high probe signals in both T and B cell subsets.^56^ In a different approach, Khan *et al.* described a copper(ii) complex of a rhodamine-azamacrocycle antagonist to bind CXCR4 in Jurkat T cells.^57^ Copper ions were reported to enhance binding by interaction with extracellular aspartate residues in CXCR4 followed by probe uptake into the cell cytoplasm.

The cannabinoid receptors 1 and 2 (CB1 and CB2) are other GPCRs commonly found in immune cells. Both CB1 and CB2 play an important immunomodulatory role, regulating cytokine production and cell migration during inflammation.^58,59^ In this context, Diaz *et al.*^60^ developed a nitrobenzoxadiazole (NBD) probe containing the CB2 inhibitor 6-methoxyisatin to target CB2 receptors in CD4+ T cells with a *K*_i_ of 387 nM and good selectivity over CB1 receptors. Interestingly, the NBD fluorophore was rationally embedded into the probe to fit in a lipophilic pocket within the CB2 receptor binding cavity. On a related project, Palomares *et al.* reported HU210-AlexaFluor488 as the first small molecule probe for CB1 receptors in immune cells.^61^ The probe was built by conjugation of the cannabinoid agonist HU210 and the AlexaFluor488 fluorophore through a 6-aminohexanoyl linker. The resulting conjugate exhibited high binding affinity for CB1 receptors over CB2 receptors, and allowed their quantification in B cells, T helper cells and cytotoxic T cells. Furthermore, the authors employed the probes to monitor the internalization of CB1 receptors in peripheral blood mononuclear cells (PBMCs) after stimulation with cannabinoid agonists.

**Fig. 4:**
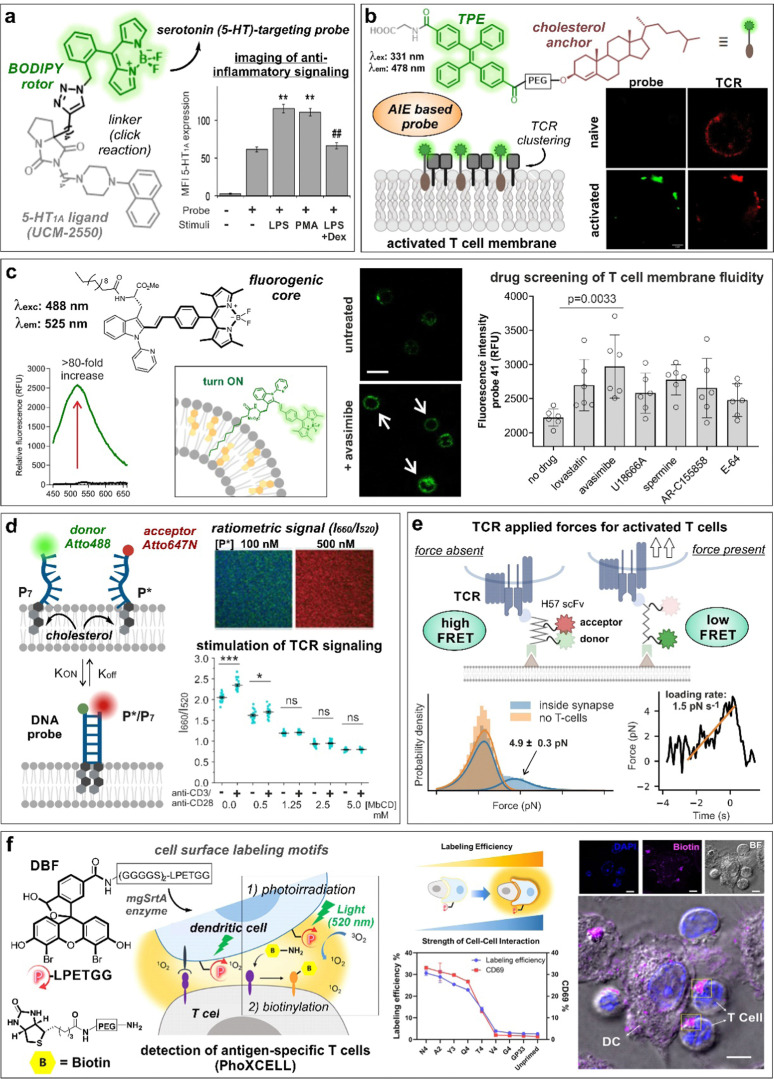
Smart probes targeting T cell specific receptors, composition and dynamics of the cell membrane. (a) UCM-2550, a BODIPY probe for imaging the serotonin receptors 5-HT1A, showed brighter staining intensity in Jurkat cells upon stimulation with LPS or PMA. The increase in fluorescence intensity with LPS stimulation was reduced in the presence of the anti-inflammatory drug dexamethasone (DEX). **, *p* < 0.01 *vs.* no stimuli; ##, *p* < 0.01 *vs.* LPS and PMA. Reproduced with permission from the American Chemical Society.^61^ (b) Structure of cholesterol-derived AIE probe, featuring tetraphenylethene (TPE) as the fluorescent moiety. The formation of probe and TCR nanoclusters in activated T cells was observed by confocal microscopy. Reproduced from ref. 74 with permission from Elsevier, copyright 2023. (c) The lipophilic tryptophan-BODIPY probe specifically inserts into the cell membrane due to its fatty chain moiety and exhibits turn-on emission in cholesterol-rich environments. Jurkat T cells incubated with avasimibe showed higher staining compared to untreated cells. (d) Schematic representation of DNA zipper mechanism of action. Increasing concentrations of FRET-acceptor (P*) led to acceptor and donor DNA strand hybridization, resulting in enhanced 660 nm emission. Activated Jurkat cells treated with DNA zipper display higher I660/I520 ratios compared to unstimulated cells. This ratio was diminished with methyl-β-cyclodextrin (MβCD) treatment in a concentration-dependent manner. ***, *p* < 0.001; ns, not significant. Reproduced from ref. 77 with permission from Wiley-VCH, copyright 2022. (e) Illustration representing the mechanism of action of FRET force sensors to measure tensile forces upon addition of activated T cells to the system. Temporal analysis of TCR-applied forces in activated T-cells was compared to gel-phase supported lipid bilayers. Reproduced with permission from the Nature Publishing Group.^83^ (f) PhoXCELL, a non-genetic platform for the detection of cell-cell interactions. Cells were labeled with DBF exploiting sortase enzyme, allowing ROS-mediated oxidation and subsequent nucleophilic biotinylation of neighbouring cells. In a model of dendritic-T cell interaction, the labeling efficiency well correlated with the expression of CD69, a marker of T cell activation. Reproduced with permission from the American Chemical Society.^84^

Serotonin receptors are GPCRs with key roles in both innate and adaptive immune responses.^62,63^ In this regard, the group of López-Rodríguez and Ortega-Gutiérrez described the preparation of a BODIPY probe for the visualization of serotonin 5-HT_1A_ receptors in immune cells (Fig. 4a).^61^ The best probe was selected from a small collection of conjugates of the 5-HT_1A_ ligand UCM-2550 to different azide/alkyne-derivatized BODIPYs. In experiments with Jurkat T cells, the authors observed an increase in fluorescence emission linked to 5-HT_1A_ receptor expression following pro-inflammatory stimuli with lipopolysaccharide (LPS) or phorbol 12-myristate 13-acetate (PMA), whereas use of the anti-inflammatory drug dexamethasone led to reduced fluorescent signals.

The chemokine receptor CCR7 guides immune cell migration and homing to lymphoid organs *via* their cognate chemokines CCL19 and CCL21.^64,65^ Recently, Legler and co-workers described an innovative method for the semi-automated production of fluorescent CCL19 and CCL21 recombinant chemokines bearing C-terminal S6-tags for site-specific labeling with fluorophores.^66^ The fluorescent chemokines (*i.e.*, CCL19-S6^Dy649P1^ and CCL21-S6^Dy649P1^) bound to CCR7-expressing cells, and CCL19-S6^Dy649P1^ was used to monitor receptor-mediated endocytosis in CD3+ T cell subpopulations isolated from PBMCs and splenocytes.

### 3.2. Fluorescent probes targeting membrane order, composition and dynamics

The signaling of GPCRs is known to be mediated by lipid–receptor, protein–receptor and receptor–receptor interactions that direct their spatial organization and clustering within the cell membrane.^67^ The activation of T lymphocytes is also linked to membrane rearrangements at both the T cell receptor and immunological synapse levels.^68^ In early studies of lipid membrane composition, the solvatochromic dye Laurdan (*i.e.*, 6-dodecanoyl-2-(*N*,*N*-dimethylamino)naphthalene) was used to visualize the formation of lipid ordered domains in the vicinity of T cell receptor activation sites and immunological synapses.^69^ Given its lipophilic character, Laurdan inserted into the phospholipid membranes and displayed a shift in its emission wavelength when found in condensed membranes (*i.e.*, from 500 nm to 440 nm). In experiments with Jurkat T cells, a remarkable increase of condensed membrane was detected upon activation of T cells with anti-CD3–coated beads. This effect was further extended by co-stimulation of CD3 and CD28 receptors. Subsequent reports rendered C-Laurdan as a derivative with improved photostability and sensitivity.^70^ With this analogue, Parmryd *et al.* could analyze the differences between the order of the plasma membrane and internal membranes in Jurkat T cells.^71^

Cholesterol is one of the main components present in the cell membrane. Several studies have linked its distribution and aggregation with T cell receptor nanocluster formation and activation of T cells.^72,73^ In this context, Zhang *et al.*^74^ developed an aggregation-induced emission (AIE)-based probe bearing a cholesterol anchor and a tetraphenylethene (TPE) dye (COOH-TPE-PEG-Chol) for the detection of T cell activation in real-time (Fig. 4b). COOH-TPE-PEG-Chol inserted into the lipid membrane *via* hydrophobic interactions and aggregated upon the formation of nanoclusters in active T cells. In experiments with CD8+ T cells isolated from blood, the authors observed that the fluorescent signals of the probe correlated well with the extent of T cell activation and with marginal emission in non-activated naïve T cells, being a promising tool to monitor T cell exhaustion in cancer tissues. In a related approach, our group reported a fluorogenic molecular rotor with turn-on fluorescence emission in response to cholesterol membrane content as a direct indicator of the T cell activation state (Fig. 4c).^75^ The probe was based on a tryptophan amino acid bearing a fatty acid chain to promote cell membrane insertion and a phenyl-BODIPY rotor with optimized fluorogenic properties. The probe displayed a viscosity sensitivity coefficient around 0.7, being among the most sensitive fluorescent rotors reported to date and was successfully implemented in a cell-based drug screen of regulators of lipid metabolism. The acyl-CoA:cholesterol acyltransferase inhibitor avasimibe, previously reported for use in combination with anti-PD-1 immunotherapies,^72^ induced the strongest activation in CD8+ T cells and resulted in the brightest fluorescence signal from the probe in flow cytometry and fluorescence microscopy experiments.

The plasma membrane is a dynamic structure where signaling events can be influenced by the interactions between protein and lipid structures.^76^ Gershenson, You and colleagues reported a fluorescent DNA-based probe to image membrane order and lipid-mediated transient interactions in the μs–ms temporal range (Fig. 4d).^77^ The probe, named DNA zipper, used a FRET activation mechanism whereby the membrane target partners were connected through short complementary DNA strands, extending the duration of transient interactions and enabling detection by ratiometric fluorescent readouts. The authors used this technology to design a cholesterol-based smart probe containing donor (Atto 488) and acceptor (Atto 647N) fluorophores on each strand and cholesterol attached at the other end of each strand. This probe enabled monitoring of the dynamic changes of membrane order in Jurkat T cells during anti-CD3/anti-CD28-stimulated T cell receptor signaling or by after incubation with the lipid disrupting agent methyl-β-cyclodextrin. In another approach, the group of Gaus reported a FRET probe (CliF) to detect intermolecular associations of neighbouring proteins during T cell receptor clustering in real-time.^78^ CliF combined a single-chain FRET pair between the yellow fluorescent protein Venus with the red fluorescent protein mCherry, a membrane anchor and the cytoplasmic domain of the subunit CD3ζ. CliF also contained the light-sensitive photoreceptor cryptochrome 2, which oligomerizes upon blue light irradiation at 488 nm, to control self-association. The authors further used CliF in Jurkat T cells to observe differences in the remodeling of CD3 clustering densities depending on the T cell stimulation method. Furthermore, the CliF technology proved also compatible with FLIM, displaying distinct lifetimes before and after clustering (*i.e.*, 1.74 ± 0.25 ns and 1.56 ± 0.29 ns, respectively).

T-cell antigen recognition drives the formation of immunological synapses, which are composed of a central TCR cluster surrounded by adhesion proteins (*e.g.*, LFA-1).^79^ Back in 2007, Kaizuka, Vale and colleagues^80^ provided a model to study the formation of immunological synapses in Jurkat T cells transfected with GFP-actin and a glass-supported lipid bilayer containing fluorescently labeled stimulatory molecules (*e.g.*, anti-CD3ε, ICAM-1) as mimics of antigen-presenting cells. This model was used to study the segregation of T cell receptors and adhesion proteins at earliest stages of synapse formation. Mechanical forces are also implicated in the discrimination of antigen peptides on ligand-engaged T cell receptors during antigen recognition.^81,82^ The group of Schütz described a FRET probe bearing a TCR-reactive antibody fragment or a peptide-loaded MHC to quantify the forces associated with T cell receptor signaling at a molecular level (Fig. 4e).^83^ The sensor was embedded within a glass-supported lipid bilayer and included a 25-mer peptide with known elastic properties, which led a decrease in FRET activation in the presence of a tensile force due to the increased distance between the two fluorophores. In another innovative approach, Li, Chen and co-workers^84^ described the quantitative detection of antigen-specific CD8+ T cell-dendritic cell interactions by means of a photocatalytic proximity cell labeling (PhoXCELL) approach (Fig. 4f). Upon irradiation (520 nm), functionalized dendritic cells with the photosensitizer dibromofluorescein generated single oxygen and promoted the biotin labeling of T cells interacting with the dendritic cells at the immunological synapse. Furthermore, this PhoXCELL strategy allowed to simultaneously detect tumor antigen-specific CD8+ and CD4+ T cells from tumor-infiltrating lymphocytes and draining lymph nodes in preclinical mouse models.

### 3.3. Fluorescent probes targeting exhaustion markers

The persistent exposure of infiltrating CD8+ T cells to tumor microenvironmental factors induces a dynamic differentiation process, so called exhaustion, characterized by the upregulation of cell membrane inhibitory receptors (*e.g.*, programmed cell death protein 1 (PD-1), cytotoxic T-lymphocyte antigen 4 (CTLA-4), T cell immunoglobulin and mucin-domain containing-3 (TIM-3)).^85,86^ In particular, the CTLA-4 signaling pathway is an important regulator of the functions of Tregs.^87^ Examples of CTLA-4-targeting probes include the detection of CTLA-4+ T cell subsets in peripheral blood and tumor tissues by means of fluorescent quantum dots coupled to CTLA-4-specific nanobodies (QDs-Nb36).^88^ Upon incubation with QDs-Nb36, polyhydroxyalkanoate-stimulated T cells displayed stronger fluorescence signals compared to untreated T cells. In another example, Kobayashi *et al.*^89^ reported an antibody-photoabsorber conjugate bearing the NIR IRDye700 fluorophore (anti-CTL-4-IRDye700) to study the local depletion of intratumoral CTLA-4 + Foxp3+ Tregs by means of photoimmunotherapy with NIR light. Blocking of CTLA4-axis led to an increase in activation and infiltration of CD8+ T cells within the TME. CD25 is a component of the IL-2 receptor known to mediate the activity of effector T cells.^90^ In another strategy, a CD25-targeted NIR photoimmunotherapy was reported to selectively deplete CD4 + CD25 + Foxp3+ Tregs within the TME, with concomitant activation of CD8+ T and NK cytotoxic effects.^91^ The authors observed >85% depletion of CD25+ Tregs using irradiation regimes that were compatible with *in vivo* experiments. Moreover, the depletion of intratumoral Tregs lasted for up to 4 days and this effect was extended to distant tumors. Despite these recent contributions, the use of fluorescent smart probes to target T cell exhaustion has been underexplored and will likely emerge in the coming years due to its significance in tumor immunology.

## 4. Targeting enzymatic activity in T cells

Enzymes are among the most common biological targets of fluorescent smart probes. The chemical adaptability of enzyme substrates, which can range from small molecules to peptides or larger proteins, to different activation mechanisms (*e.g.*, FRET, PeT, ICT) makes them attractive scaffolds for the construction of activatable probes that can report the functional state of enzymes. Among the different families of enzymes found in lymphocytes, granzymes are serine proteases that are primarily produced and secreted by cytotoxic T cells and natural killer (NK) cells as part of the immune response to viral infections and cancer cells.^92^ Granzymes play a key role in programmed cell death by cleaving specific proteins in the target cells (*e.g.*, pro-caspases) and initiating a cascade of events that results in cell death.^93^ In view of this, granzyme-targeting smart fluorescent probes have been considered as an effective approach to detect and visualize T cell and NK cell activity under physiological conditions.

There are 5 granzymes in humans (*i.e.*, granzymes A, B, H, K, M), with granzyme B being the most targeted of all. Differential levels of granzyme B (GzmB) have been associated with multiple disorders, from cancer to infection or auto-immune diseases. Notably, GzmB was found to be overexpressed in biological fluids of patients affected by chronic inflammatory diseases, including synovial fluid in rheumatoid arthritis and broncho-alveolar lavage from asthma patients. GzmB is also known to play a role in regulating inflammation and the host immune response, therefore being a potential biomarker of disease progression and response to therapy.^94^

### 4.1. Activity-based and PET probes for granzymes

The proteolytic activity of granzymes prompted the discovery of peptide sequences that could be specifically recognised by the different enzyme subtypes. Seminal work in this field was reported by the groups of Nicholson and Craik, who investigated the substrate specificity of GzmA and GzmB to prepare peptide-based inhibitors.^95,96^ For instance, the combination of GzmA- and GzmB-specific substrate sequences (IGNR and IEPD, respectively) with a diphenyl phosphonate warhead led to highly specific inhibitors. These probes were exploited to gain insight on granzyme biology and helped in determining the roles of GzmA and GzmB in NK cell-mediated target cell lysis.^97^ In addition to their potential use as therapeutics, the discovery of selective GzmB inhibitors opened avenues to the molecular design of radiolabeled agents for PET imaging of T cell activity. In this case, the covalent binding of radiotracers to the active site results in preferential accumulation of the probe in tissues with high GzmB activity. Several examples of such inhibitor-based molecular probes have been described in immuno-oncology studies to predict response to checkpoint inhibitors in mouse preclinical models of cancer.^98–100^ For instance, Craik and colleagues designed a smart PET probe based on a restricted interaction peptide in which a radiolabeled antimicrobial peptide was connected to a caged sequence by means of a GzmB cleavable linker.^101^ Upon cleavage of the sequence mediated by GzmB, the radiolabeled antimicrobial peptide was liberated and able to accumulate in adjacent cell membranes. This smart probe was successfully used to monitor GzmB activity in three different syngeneic mouse models of cancer and also in a model of pulmonary inflammation.

### 4.2. Nanoparticle-based imaging of GzmB activity

In parallel, the discovery of selective sequences for GzmB also accelerated the design of smart probes that could generate a detectable optical readouts after enzymatic cleavage.^102^ In this case, the contrast would be generated by the release of a unique optical fingerprint (*e.g.*, bright luminescence signal, ratiometric change in emission wavelengths) after the smart probe would have reacted with the active form of the enzyme. Some of the first designs considered the construction of smart nanomaterials that incorporated GzmB-reactive sequences. For instance, Libby and co-workers reported a GzmB-targeting nanoprobe using a poly-lysine graft copolymer scaffold.^103^ Briefly, the authors modified the murine-specific sequence GIEFDSGGC with a Cy5.5 fluorophore and generated a self-quenched fluorogenic polymer that released bright emission after cleavage by the enzyme. Their nanoprobe displayed ∼4-fold fluorescence increase upon reaction with mouse GzmB and was used *in vivo* to image cytotoxic CD8+ T cells in the heart of mice undergoing lymphocyte-mediated myocarditis *via* fluorescence molecular tomography.

A similar self-quenched nanoprobe was reported by the group of Kwong for non-invasive detection of acute transplant rejection, where recipient T cells secrete high levels of GzmB during the onset of rejection.^104^ In this case, the authors conjugated a NIR-labeled GzmB peptide substrate (after screening of several sequences and coupling to IRDye800CW) to PEGylated iron oxide nanoparticles for efficient biodistribution and extended circulation. The smart nanoprobes were administered systemically in mouse models of skin graft rejection, where their accumulation in allograft tissues and subsequent cleavage by GzmB resulted in the release of the fluorescent peptide to the urine.

Self-quenching mechanisms can be also integrated into polymers that generate ratiometric signals in a GzmB-dependent manner. A good example of this approach was reported by the group of Pu, who exploited the dual fluorescence and photoacoustic properties of IRDye800CW to synthesize self-assembled semiconducting polymers containing NIR-labeled peptide substrates.^105^ The cleavage of the peptides led to reduced fluorescence and photoacoustic signals but unchanged signals from the semiconducting polymer, thus producing a direct ratiometric readout of GzmB activity.

In addition to self-quenching nanomaterials, smart nanoprobes for monitoring GzmB activity can be also generated using alternative reporting mechanisms, such as FRET pairs. Kulkarni *et al.* recently described smart nanoprobes where FRET-paired peptide substrates (two FRET pairs were employed: in the visible range, fluorescein as the fluorophore and QSY-7 as the quencher; in the NIR range, DyLight755 as the fluorophore and DyLight766Q as the quencher) were conjugated to a poly(isobutylene-*alt*-maleic anhydride) scaffold and subsequently coupled to anti-PD-L1.^106^ The inhibition of PD-1/PD-L1 induced T cell-mediated GzmB release and enabled real-time imaging of immunotherapy response in tumor-bearing mice, being able to distinguish between highly responsive and poorly responsive tumors.

**Fig. 5:**
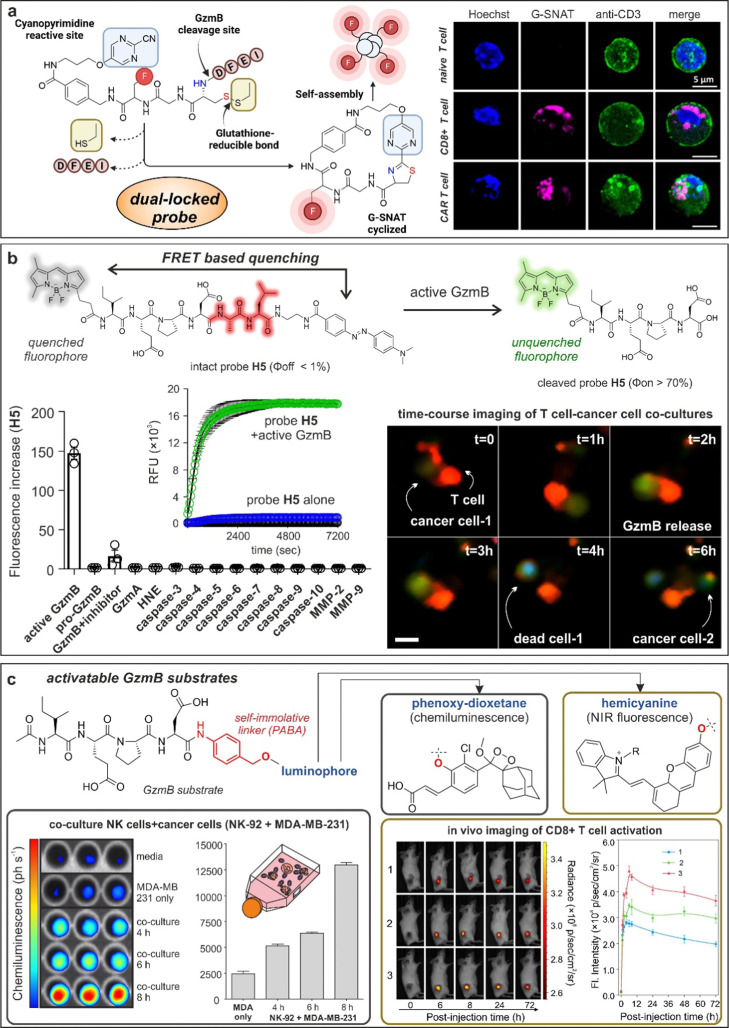
Granzyme-activatable probes exploiting different activation modalities. (a) Dual-locked probes. Illustration of the chemical structure and cyclization mechanism of G-SNAT probe leading to self-aggregation and enhanced emission. Confocal imaging of T cells incubated with G-SNAT showed fluorescent emission only in stimulated T cells and CAR T cells. Reproduced with permission from the American Chemical Society.^109^ (b) FRET quenching. Chemical structure and mechanism of activation of probe H5 featuring a BODIPY fluorophore and Dabcyl quencher. H5 showed exquisite selectivity for GzmB over other proteases and fast kinetic of activation. Time-course live cell imaging allowed the visualization of GzmB activity (green) in a cancer cell-T cell co-culture model. (c) Activatable granzyme substrates. The granzyme-specific sequence IEPD was conjugated to chemiluminescent phenoxy-dioxetanes or NIR fluorophore hemicyanines by means of a self-immolative linker. Incubation of chemiluminescent probe with a co-culture model of NK and cancerous cells (MDA-MB-231) led to time-dependent photon emission. *In vivo* imaging of T cell activation in tumor-bearing mice exploiting GzmB responsive hemicyanine sensor and quantification of the NIR fluorescence signal in the tumor site as a function of post-injection time. Reproduced with permission from the American Chemical Society.^115^

One emerging chemical strategy to increase the signal-to-noise ratios of smart probes is *via* the preparation of AND-gate or dual-locked probes, which require two or more sequential degrees of activation to produce detectable signals.^107^ The group of Liang built on this concept to prepare dual-quenched nanoparticles for imaging T cell activity *in vivo*.^108^ In this work, the authors designed a Cy5.5-labeled GzmB-reactive sequence with a 2-cyanobenzothiazole tag that could form a cyclic dimeric product and spontaneously self-assemble into silent GzmB-responsive nanoparticles where the fluorescence signals were quenched through (1) intermolecular hydrophobic interaction and (2) π–π stacking. The authors demonstrated that GzmB cleavage led to nanoparticle disassembly and turn-on fluorescence emission, allowing *in vivo* imaging of active GzmB in tumor-bearing mice. Interestingly, nanoprobe self-assembly can be also used as a chemical strategy to turn-on fluorescence emission. An excellent example was recently reported by the team of Rao, who designed G-SNAT as a GzmB-activatable self-assembly smart probe for the assessment of cytotoxic T lymphocyte-mediated cancer cell killing.^109^ G-SNAT incorporated a 2-cyanopyrimidine group in close proximity to a GzmB-responsive IEFD peptide so that intramolecular cyclization occurred after (1) GzmB cleavage and (2) reduction of the disulfide bond by intracellular glutathione (Fig. 5a). The resulting macrocycles were susceptible to intermolecular interactions that triggered *in situ* nanoaggregation and concomitant fluorescence release. Finally, the authors employed G-SNAT in a preclinical model of cancer to monitor the cytotoxic activity of CAR T cells and checkpoint blockade therapies in mice.

### 4.3. Chemiluminescent and fluorescent activatable probes for granzymes

Although nanomaterials are convenient architectures to amplify the activation of enzyme-responsive probes and obtain high signal-to-noise ratios, they are hard to translate to clinical studies because of their often-slow kinetics and manufacturing challenges.^110^ To this end, several groups have described simpler smart probes to image T cell activity. FRET-compatible peptide substrates are one of the most common structures for the rapid detection of active enzymes.^111^ These often involved short peptide sequences flanked by fluorophore:quencher pairs or donor:acceptor fluorophores;^112^ however, their chemical optimization to achieve high selectivity and reactivity is not straightforward. Our group reported the rational design of a unique FRET-based hexapeptide H5, which shows an unprecedented *k*_cat_/*K*_M_ ratio of 1.2 × 10^7^ M^−1^ s^−1^ and a limit of detection of 6 pM, being able to detect physiological levels of GzmB in human tissues and fluids (Fig. 5b).^113^ Notably, molecular dynamic simulations highlighted an alternative binding mode for H5 to human GzmB, which could facilitate the design of additional smart probes and therapeutics. The high reactivity and selectivity of the probe H5 enabled real-time detection of T cell activity in biopsies from lung cancer patients and adaptability to image-based phenotypic screens, which identified for the first time an immune-mediated anticancer activity for the AKT kinase inhibitor AZD5363.

**Fig. 6:**
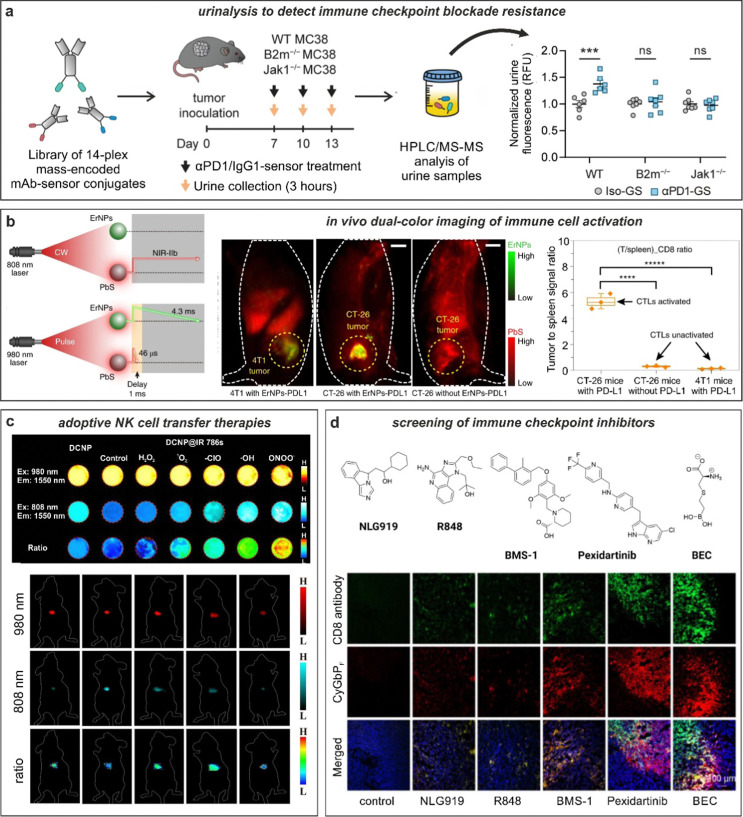
Smart fluorescent probes for the evaluation of anti-cancer immunotherapies. (a) GzmB fluorescent substrates were conjugated to anti-PD-1 (αPD-1-GS) or isotype (iso-GS) antibodies and administered to wild-type or mutated tumor-bearing mice to monitor anti-tumor responses. Analysis of fluorescence emission in the urine samples allowed for the detection of anti-tumor response in wild-type but not in immunotherapy-resistant mice. ***, *P* < 0.001; NS, not significant. Reproduced with permission from the Nature Publishing Group.^133^ (b) *In vivo* tracking of T cells exploiting quantum dots (PbS) and rare-earth nanoparticles (ErNPs). NIR imaging in tumor-bearing mice showed strong co-localization of ErNPs-aPDL1 and PbS-aCD8 in CT26 tumors, whereas weak signals were detected in 4T1 tumors. Reproduced with permission from the Nature Publishing Group.^134^ (c) *In vivo* evaluation of cell viability in adoptive cell-based immunotherapy. Down-conversion nanoparticles (DCNP) decorated with IR786 dye are imaged in presence of different ROS species. *In vivo* adoptive cell viability evaluation using ratiometric NIR-II measurements in tumor bearing mice after 1 h, 1 d, 3 d, 5 d, and 11 d, respectively. Reproduced from ref. 138 with permission from Wiley-VCH, copyright 2021. (d) Fluorescence-based drug screening. Chemical structures of different drug candidates for the stimulation of antitumoral responses. Immunotherapeutics were injected into tumor-bearing mice followed by administration of the NIR probe, CyGBP_F_. Tumor biopsies were harvested, and the tissue imaged to evaluate the extent of drug-induced T cell activation. Reproduced with permission from the American Chemical Society.^115^

One potential disadvantage of FRET probes composed of large and hydrophobic fluorophores and quenchers -particularly those active in the NIR spectral range- is their limited water solubility, which can hinder some biological applications, such as *in vivo* studies.^114^ Fluorogenic substrates can mitigate this limitation by employing a single fluorescent structure that responds to enzymatic cleavage. Pu and co-workers described some of the first NIR fluorogenic probes for mouse and human GzmB.^115^ The authors used a self-immolative *p*-amino benzyl alcohol linker to connect the peptides IEFD (mouse) and IEPD (human) to a hemicyanine reporter (Fig. 5c and 6d). Notably, the conjugation of the hemicyanine core *via* the phenol group quenched the fluorescence emission of the NIR dye, which was only restored after GzmB cleavage. The mouse-reactive probe was systemically administered in tumor-bearing mice to measure *in vivo* GzmB signals, which correlated with the location of CD8+ cytotoxic T lymphocytes in tumor tissues.

A critical aspect to optimize in GzmB-activatable probes is their selectivity over other enzymes that cleave after an aspartic acid residue, such as caspases. Kasperkiewicz *et al.* addressed this point by producing libraries of peptide sequences that included different unnatural amino acids.^116^ Notably, the incorporation of amino acids like octahydroindole-2-carboxylic acid (Oic) and norvaline (Nva) led to GzmB-selective probes with *k*_cat_/*K*_M_ values in the 10^5^ M^−1^ s^−1^ range. Furthermore, once optimal sequences were identified, the authors prepared diphenylphosphonate inhibitor-based probes -for cell lysate analysis- and FRET-based smart probes -for live cell microscopy- and employed them to image the localization and activity of GzmB in a range of NK cells.

Whereas most optical probes to study GzmB activity in T cells and NK cells are based on fluorescent structures, recent studies have reported the preparation of activatable chemi- and bioluminescent probes. Chemi- and bioluminophores are advantageous in that, unlike fluorophores, they do not require excitation with an external light source, which can result in lower background signals and increased turn-on ratios.^117^ In the last years, the field of chemiluminescence imaging has been boosted by the seminal discovery of Shabat and co-workers with biocompatible 1,2-dioxetanes.^118^ These structures are modular and versatile scaffolds for the construction of smart probes, including enzyme-activatable probes.^119^ Together with Kitamura and Shabat, our group reported the first GzmB-activatable chemiluminescent probe.^120^ Briefly, the probe consisted of the reactive IEPD sequence conjugated to a 1,2-dioxetane chemiluminophore *via* a self-immolative linker (Fig. 5c). Notably, this approach led to increased signal-to-noise ratios over fluorescent counterparts (*i.e.*, Ac-IEPD-aminomethylcoumarin), which were exploited to image the anti-cancer activity of human NK cells in co-cultures and in mouse xenografts. The group of Rao reported an analogous design to prepare GBLI-2 as the first bioluminescent smart probe for mouse GzmB detection.^121^ In this case, the authors conjugated the IEFD sequence to luciferin with the same *p*-aminobenzyl alcohol linker. GBLI-2 was used to predict the efficacy of immunotherapies by longitudinal imaging of CD8+ T cell activity in mouse models *in vivo*.

### 4.4. Imaging of GzmA activity

In addition to GzmB, recent efforts to generate T-cell targeted enzymatic smart probes have been focused on the protein GzmA. GzmA is another important member of the granzyme family present in the cytolytic granules from CTLs and NK cells.^122^ Although its biological function remains controversial, GzmA is reported to cause the death of the target cell by inducing reactive oxygen species (ROS) production and by activating different pro-apoptotic proteins compared to GzmB.^123^ Initial approaches to detect GzmA activity have exploited some of the molecular designs that had been previously successful for GzmB targeting. For instance, our group recently reported the first NIR fluorogenic probes for monitoring GzmA activity.^124^ This family of probes consisted of GzmA-reactive tetrapeptides (IGNR for human GzmA, GFFR for mouse GzmA) and a fluorogenic hemicyanine that turned on upon selective enzymatic cleavage. The probes displayed good selectivity over granzymes and proteases, as shown in experiments with tissue lysates from GzmA knock-out mice. The fast reactivity of the probes also enabled real-time imaging of antigen-driven recognition of mouse cancer cells by cytotoxic T lymphocytes where active GzmA and active GzmB were visualized simultaneously by combining the NIR GzmA probe and the previously reported green fluorescent H5 smart probe for GzmB. Future efforts in this area will involve the design of more reactive and selective chemical structures over other members of the tryptase-like family of proteases. To this end, the work by Kasperkiewicz and co-workers with the screening of novel peptide sequences to identify more selective GzmA substrates^125^ will open avenues to generate the next generation of fluorescent smart probes to study T cell function under physiological conditions.

## 5. Imaging of anti-cancer immunotherapies

The advent of immunotherapy has revolutionized the treatment of cancer, providing a level of effectiveness that was rarely achievable with traditional chemotherapeutic agents. Nowadays, many first-line treatments of neoplastic diseases are based on immunotherapies with good clinical efficacy.^126^ Despite several successful examples, anti-cancer immunotherapies do not benefit all patients and better ways to optimize regimes (*e.g.*, combination therapies) and dosages are urgently needed. Given that the efficacy of anti-cancer immunotherapies is closely related to the infiltration of cytotoxic cells (*e.g.*, T lymphocytes, NK cells), multiple approaches to track their *in vivo* localization and function have been described.

One of the most conventional methods for *in vivo* imaging of therapeutic response is the use of transgenic mouse models containing optical reporter genes (*i.e.*, fluorescent proteins or enzymes like luciferases that produce bioluminescence readouts) in specific cells. The incorporation of such targeting vectors enables targeted imaging at multiple levels, from fluorescence microscopy cell-based assays for drug screens to intravital and whole-body imaging in intact organisms that correlate T cell location and therapy outcome. The main advantage of optical reporter genes is that their insertion at a specific locus result in selective targeting of subpopulations of T cells and the ability to longitudinally track them over time, helping to investigate the effect of anti-cancer immunotherapies. The group of Mezzanotte recently reported a summary of transgenic mouse models for fluorescence and bioluminescence imaging of T cells.^127^ These models range from BLITC mice for generic monitoring of T cell localization (*e.g.*, infiltration of T cells into tumors) to Foxp3 reporter mice to detect subsets of T cells, such as T regs. On the other hand, the main shortcoming of transgenic mouse models is their limited translatability to optical imaging in humans, which has encouraged the chemical development of molecular imaging agents that can similarly target subpopulations of immune cells. For instance, our group has demonstrated that the combination of small proteins (*e.g.*, chemokines) with pH or protease-activatable fluorophores^128^ can be used to target defined subpopulations of immune cells that could not be monitored with a single optical reporter.^129^

### 5.1. Antibody-based probes for imaging of efficacy markers

Capturing the dynamic information rising from the interactions between cancer cells and T cells requires the selection of suitable biomarkers,^130^ with radiolabeled PET probes being in a more advanced translational state, as recently reviewed by Whitney and Aarntzen.^131^ The molecular targets for T cells are relatively diverse, from generic surface receptors (*e.g.*, CD4, CD8) to T cell activation markers (*e.g.*, PD-1, LAG-3, OX40, IL-2) or more recently, metabolic markers (*e.g.*, ^18^F-labeled amino acids and nucleobases). However, one shortcoming of PET radiotracers is their limited multiplexity, which has prompted the design of multicolour optical technologies for immunohistochemistry to examine T cell phenotypes with high spatial resolution. In the context of anti-cancer immunotherapies, Vasaturo and Galon have reported methods using fluorescence imaging of fixed tumor tissue biopsies with panels of antibody-based probes to quantify the expression and co-localization of multiple biomarkers (*e.g.*, CD8, PD-L1) and their adaptation to image analysis and digital pathology.^28^ Antibodies (*e.g.*, anti-PD-1) conjugated to bright NIR dyes such as IRDye800CW also hold promise as imaging agents forfluorescence-guided tumor resection during surgery.^132^ In the future, these platforms could be integrated into existing patient management routes and accelerate the stratification of patients and the selection of immune checkpoint inhibitors in a more personalized manner.

Antibodies binding to markers of T cell activation (*e.g.*, PD-1) can be also chemically tuned to generate smart probes with enhanced selectivity and sensitivity. One excellent example was reported by the group of Kwong with the chemical design of anti-PD-1 based sensors to monitor response to immune checkpoint blockade therapy.^133^ In this work, the authors conjugated (*e.g.*, *via* standard lysine derivatization) protease substrates -for GzmB and others- to anti-PD-1 antibodies so that specific proteolysis readouts from responder mice (but not from treatment-resistant subjects) was obtained. The authors included mass barcodes into the substrates that allowed simultaneous measurements of mass spectrometry readouts in urine samples (Fig. 6a). Notably, whereas a GzmB-targeted sensor lacked the ability to differentiate between mechanisms of resistance, the mass fingerprints obtained from a library of anti-PD-1 barcoded probes for multiple proteases was able to detect early antitumor responses and discern between resistance mechanisms.

Antibodies have been also conjugated to nanoparticles with optimized properties for *in vivo* imaging in preclinical models. The group of Dai reported metal-based nanoparticles for real-time imaging of anti-cancer immunotherapy.^134^ The authors synthesized bright erbium nanoparticles emitting in the long NIR-IIb window, which spans from 1500 nm to 1700 nm, where tissue background is very low and signal-to-noise ratios can be maximized (Fig. 6b).^135^ The nanoparticles were conjugated to anti-PD-L1 antibodies for targeting and used in combination with T cell markers (in this case, anti-CD8-derivatized quantum dots) to simultaneously monitor T cell location and T cell activation in mouse models of colorectal cancer. The chemical properties of these nanoparticles were optimized to enhance detection, including long emission wavelengths for deep tissue penetration (*i.e.*, subcentimeter range), high signal-to-noise ratios (*e.g.*, around 40), good biocompatibility *via* hydrophilic coating and long fluorescence lifetimes (*i.e.*, close to 5 ms) for multiplexed imaging in the NIR-II window. In a follow-up work, the same group further optimized the cross-linked coating on the surface of quantum dots to maximise biocompatibility and excretion after intravenous administration.^136^ In this case, the authors coated quantum dots with amphiphilic polymeric layers containing branched PEG and linear polyacrylic acid and modified them with anti-CD8 diabodies for T cell targeting. The good biocompatibility of these particles was exploited for NIR imaging of cytotoxic T lymphocytes in response to anti-PD-L1 immunotherapy in mice using light sheet microscopy with very high signal-to-noise ratios (*i.e.*, >150) and cellular resolution.

### 5.2. Probes for *in vivo* detection of cell viability

Alternative nanoprobe designs for imaging response to immunotherapy can generate contrast based on activatable reporters rather than antibody-based accumulation. The groups of Pu and Zhang reported semiconducting polymer nanoreporters including chemiluminophores that were sensitive to superoxide anion for *in vivo* imaging of T cell activation.^137^ Briefly, the nanoconstructs passively accumulated into tumors in mice, and included a caged dioxetane for chemiluminescence resonance energy transfer. Using this approach, regions of low and high superoxide levels -in principle, related to weak and strong T cell activation states- could be discerned. For instance, in areas with low levels of superoxide, only fluorescence signals from the semiconducting polymer would be detected whereas high levels of superoxide decaged the dioxetane moiety, resulting in both bright chemiluminescence and fluorescence readouts. Given the tunability and versatility of phenoxydioxetane chemiluminophores, this molecular design could represent a modular approach to construct smart probes for monitoring T cell function in response to anti-cancer immunotherapies.

ROS can be also used as biomarkers for cell viability, which is an essential parameter in adoptive cell transfer immunotherapies where cancer-attacking cells (*e.g.*, NK cells) are administered intravenously to kill tumors more effectively. The groups of Liu and Song recently reported a strategy to measure the viability of adoptive transferred in NK cells using ratiometric fluorescence imaging.^138^ The authors prepared lanthanide-based nanoparticles with inherent NIR-II emission that were coated with the ROS-sensitive NIR-I dye IR786 and used them to pre-label adoptively transferred NK cells (Fig. 6c). Interestingly, the high intracellular ROS levels in dead NK cells (but not in NK viable cells) led to an increase of the fluorescence signal under excitation at 808 nm due to degradation of the IR786 dye and different ratiometric response signals. The differential readout of NK cell viability was further employed to demonstrate that the administration of cytokines (*e.g.*, IL-2, IL-15, and IL-21) improved the *in vivo* survival of NK cells and led to better anti-cancer outcomes in a mouse model of hepatocellular carcinoma.

The success of adoptive T cell transfer anti-cancer therapies heavily relies on the ability of T cells to target and retain within tumors. Smaller chemical constructs, such as NIR dyes, have found good use as labeling agents for tracing the *in vivo* location of T cells following adoptive transfer. For example, Zweit *et al.* used the non-toxic and membrane-targeting dye 1,1-dioctadecyltetramethyl indotricarbocyanine iodide (DiR) to pre-label T lymphocytes before they were administered intravenously into tumor-bearing mice.^139^ Because the T lymphocytes were isolated from draining lymph nodes of 4T1-sensitized mice and activated *in vitro*, they specifically accumulated in 4T1 tumors grown in the recipient mice. The authors performed *in vivo* fluorescence imaging to detect NIR emission from the labeled T cells and found that tumor accumulation peaked at 6 days and signals being detectable for up to 3 weeks after injection. In order to overcome the limited penetration depth of NIR fluorophores, recent studies have also explored photoacoustic agents, which can provide signals up to 1 cm in depth, and used them to pre-label T cells (*e.g.*, with the NIR-797 dye)^31^ and monitor their location in tumor-bearing mice. In addition to increased depth penetration, another important feature of PA imaging is the possibility to acquire label-free anatomical information from tumors *in vivo*,^140^ which can provide further insights into the efficacy of anti-cancer immunotherapies. One recent example was reported by the group of Ntziachristos, who used a combination of PA imaging modalities to visualize solid tumors receiving adoptive T cell transfer therapy and were able to monitor optical absorption changes in the tumor mass associated with blood vessel collapse during tumor rejection.^141^

### 5.3. Other imaging modalities and phototheranostics

Finally, some of the latest molecular constructs for the examination T cell-based anti-cancer immunotherapies have considered more sophisticated chemical designs with capabilities beyond optical imaging. Some examples include nanoparticles for tracking of T cells using multimodal imaging (*e.g.*, *via* fluorescence and computed tomography) or theranostic probes for simultaneous detection and therapeutic delivery.^142^ Regarding the latter, Yoon and co-workers described nanoparticles based on heavy-atom-free BODIPY fluorophores that remained non-photoactive until disassembled by interaction with albumin.^143^ The authors showed the application of these constructs for both *in vivo* tumor detection (*e.g.*, *via* PA imaging) and photoablation (*e.g.*, *via* photodynamic and photothermal therapy) alongside its synergistic combination with immune checkpoint inhibitors. Also, the group of Dai reported phototheranostic nanoprobes with capacity for NIR-II fluorescence and PA imaging as well as photothermal therapy.^144^ Of note, this design considered the encapsulation of drugs (*e.g.*, ferroptosis inducers), which resulted in strong T cell reinvigoration and anti-cancer immunity.

## 6. Conclusions and outlook

Anti-cancer immunotherapies, from immune checkpoint blockade antibodies to CAR T cells, are emerging strategies to reprogram the cytotoxic activity of the host immune system (*e.g.*, T cells and NK cells) against tumors. In this article, we reviewed recent advances in the chemical design of smart probes to image T cells, including different molecular architectures and numerous biological targets and applications. Initial designs were focused on tracking the migration and proliferation of T cells to sites of action (*e.g.*, tumors, inflamed tissues), whereas recent designs have shifted towards distinguishing between different T cell subpopulations and metabolic states. For instance, the stimulation of T cells leads to changes in the lipid composition and dynamics of the plasma membrane, which can be sensed by smart probes containing environmentally sensitive fluorophores. Other important biomarkers involve the secretion of proteolytic enzymes (*e.g.*, GzmA and GzmB), which are key effector molecules in cytotoxic T cells and can be used for real-time monitoring of anti-cancer immunotherapy efficacy. In parallel to the design of smart probes for stimulated T cells, there is a need for new complementary probes that can detect T cell exhaustion, a process that influences the efficacy of anti-cancer treatments in the tumor microenvironment, or that combine multiple biomarkers in one single smart probe (*e.g.*, AND-gate probes, dual-lock probes) to target subpopulations of T cells (*e.g.*, Tregs). The development of new activatable chemical moieties will accelerate the synthesis of the next generation of smart probes for T cells. The chemical diversity in this space is large, and ranges from fluorescent amino acids and biomarker-activatable fluorophores to NIR scaffolds with emission in the NIR-II window or compatibility with photoacoustic imaging. Given the biological complexity of tissue microenvironments (*e.g.*, tumors) and the broad range of T cell subpopulations with variable functional readouts, we envisage that fluorescent reporters coupled to T cell specific biomolecules (*e.g.*, peptides, chemokines, antibodies) will produce a new toolbox of smart probes for T cells to interrogate T cell biology in multiple disease contexts. Finally, the translation of T cell probes towards the clinic holds huge potential for improving the diagnosis of inflammatory diseases and the personalized optimization of anti-cancer immunotherapies. Examples of these applications may include the integration of smart probes into existing clinical imaging modalities to track the presence of T cells directly in humans^145^ as well as their adaptation for the analysis of biosamples (*e.g.*, urine, tissue biopsies) to obtain complementary readouts to those provided by already existing procedures (*e.g.*, ELISA assays, immunohistochemistry).

## Conflicts of interest

The authors declare no conflicts of interest.

## Supplementary Material
